# Job Satisfaction and Health Problems Among Cabin Crew: The Mediating Role of Burnout

**DOI:** 10.3390/healthcare14040473

**Published:** 2026-02-13

**Authors:** Dailet Fredes-Collarte, Víctor Olivares-Faúndez, José Carlos Sánchez-García, Francisco Ganga Contreras, Jenniffer Peralta Montecinos, Jeamsie Herrera Parraguez

**Affiliations:** 1 Research Group on Occupational Health and Organizational Development, Department of Social Sciences, University of Tarapacá, Iquique 1100000, Chile; 2 Research Group on Occupational Health and Organizational Development, Psychology School, University Autonomy de Chile, Santiago 7500000, Chile; 3 Department of Social Psychology and Anthropology, University of Salamanca, 37008 Salamanca, Spain; jsanchez@usal.es; 4 Facultad de Educación y Humanidades, Universidad de Tarapacá, Arica 1000007, Chile; franciscoganga@academicos.uta.cl; 5 Research Group on Occupational Health and Organizational Development, University of Tarapacá, Arica 1000007, Chile; jperalta@academicos.uta.cl; 6 Faculty of Administration and Business, University of Tarapacá, Iquique 1100000, Chile; jherrera@academicos.uta.cl

**Keywords:** burnout, job satisfaction, heath problems, structural equations, cabin crew

## Abstract

Background/Objectives: The aviation sector is characterized by high-density flight operations and chronic stressors that compromise worker health. This study focuses on burnout syndrome as a multidimensional phenomenon resulting from the interaction between high emotional demands and personal resources. The primary objective was to analyze the relationship between job satisfaction and health problems among cabin crew members, testing a structural model where burnout—specifically its dimensions of enthusiasm toward the job, psychological strain, indolence, and guilt—acts as a mediating factor. Methods: A quantitative, cross-sectional design was implemented with a sample of 732 cabin crew members from an international airline. Participants completed the Spanish Burnout Inventory (SBI) and the UNIPSICO subscales for job satisfaction and psychosomatic problems. Data was processed using Structural Equation Modeling (SEM) to evaluate the hypothesized interdependent relationships and global model fit. Results: The structural model demonstrated an acceptable fit (CFI = 0.890; RMSEA = 0.056), confirming that job satisfaction is positively related to enthusiasm toward the job and inversely associated with psychological strain. All burnout dimensions were significantly linked to health outcomes; notably, guilt was identified as a critical mediator between indolence and psychosomatic problems. Conclusions: The findings underscore burnout as an insidiously progressive process that mediates the deterioration of cabin crew health. The study highlights guilt as a determining factor in the syndrome’s severity. Consequently, preventive organizational strategies must move beyond general fatigue management to include emotional labor training and early diagnosis of psychosocial risks to preserve operational safety and crew well-being.

## 1. Introduction

In recent decades, various stressors have been identified within work environments in the aviation sector. Authors such as Alam [1] associate this complexity with technostress derived from globalization, a process that has intensified international, regional, and national connections and, consequently, the job demands placed on workers in the sector. This context, along with new work configurations, has transformed the employee experience [2]. While these changes generate opportunities, they also establish tensions stemming from organizational pressure for efficiency and effectiveness, requiring a greater capacity for adaptability and personal development to face the challenges of a highly volatile environment [3]. In this sense, transformations in the world of work have impacted worker health, manifesting through phenomena such as occupational stress and the deterioration of interpersonal relationships. These elements act as psychosocial risk factors that influence socialization processes and, ultimately, compromise occupational well-being [4].

To explore these risks, the present study focuses on Burnout Syndrome. Burnout is defined as a psychological response to chronic occupational stress [5]. It is a phenomenon linked to psychosocial risks of high organizational concern, as it generates emotional and interpersonal exhaustion that persistently affects the worker’s social and family spheres [6]. Along these lines, the World Health Organization (WHO) defines it as a syndrome resulting from chronic workplace stress that has not been successfully managed [7]. This phenomenon has more than just individual consequences; authors such as Gil-Monte [2] point out negative repercussions for the organization, such as increased absenteeism and staff turnover.

Beyond the impact on organizational dynamics, prospective scientific evidence underscores that burnout acts as a predictor of various physical and psychological pathologies [8]. This health deterioration culminates in a detrimental cycle that affects professional performance through presenteeism and absenteeism, impairing customer service [9], especially in human service industries where emotional demands and interpersonal interactions are high [10].

Research in the aviation sector has primarily focused on cabin crew due to their exposure to critical work environments. Various studies have evidenced the prevalence of physical and mental exhaustion in this group, manifested by high levels of fatigue, sleep problems, and anxiety [11]. These indicators result from prolonged exposure to chronic stressors derived from constant interaction with passengers [12]. A determining factor in the configuration of burnout in this context is emotional dissonance. This arises from the organizational demand to manage one’s own emotions to meet service standards [13]. Furthermore, it has been observed that these demands inherent to the aviation environment permeate the private sphere, significantly affecting the work–family interface [14].

As Maslach [15] points out, burnout is defined as a process of psychological erosion that is insidious and gradual. It is insidious because its initial manifestations are subtle and often masked by the professional standards required in the aviation sector, making early detection difficult. It is also a gradual process, as it develops through the chronic accumulation of residual fatigue and operational stressors. This progression is consistent with the work of cabin crew, whose constant exposure to emotional dissonance and flight environment demands does not trigger a sudden crisis, but rather a silent and progressive depletion of personal resources over time.

### 1.1. Job Satisfaction and Burnout Among Cabin Crew

In close relation to health and family life impacts, the burnout phenomenon directly affects a worker’s attitude toward their labor, specifically their job satisfaction. Historically, this has been conceptualized as a positive or pleasant emotional state resulting from the appraisal of one’s work. In the aviation sector, a negative relationship between burnout and job satisfaction has been identified [16]. In this regard, studies conducted with cabin crew in South Korea have confirmed that high levels of exhaustion significantly predict lower satisfaction with the work environment [17]. This evidence is reinforced by research in Taiwan, which shows that while emotional exhaustion erodes attitudinal well-being, personal accomplishment (enthusiasm toward the job) fosters optimal levels of satisfaction [18]. In this sense, evidence indicates that job satisfaction and burnout are inversely related constructs within the professional experience of flight attendants [16].

### 1.2. Health Problems and Burnout in Cabin Crew

Accumulated evidence in the aviation sector, as in other industries, demonstrates that burnout is not only a psychological phenomenon; it also manifests in physical symptoms and operational consequences.

Firstly, chronic fatigue and sleep disturbances are the most prevalent indicators. Various studies report abnormal fatigue levels in more than 60% of participants, along with a high incidence of daytime sleepiness and insomnia derived from chronodisruption (shift work and jet lag) [19,20,21,22].

Burnout has also established itself as a predictor of physical morbidity. Recent meta-analyses and cohort studies in European airlines link this syndrome to a wide range of somatic disorders, including gastrointestinal conditions, headaches, palpitations, and musculoskeletal pain [23,24,25]. Significant gender differences have been observed in this regard, with female crew members reporting greater reactivity to musculoskeletal pain compared to their male counterparts [23,26]. Furthermore, the literature suggests that the chronic stress underlying burnout could act as a risk factor for long-term metabolic diseases, such as Type 2 diabetes [8].

The impact of burnout transcends individual health and compromises operational safety. Research in diverse contexts, such as Taiwan and China, confirms that the deterioration of mental health, characterized by anxiety, hostility, and depression, translates directly into lower job performance and increased turnover intention [8,13,25,27,28,29].

### 1.3. Burnout Model

Although the scientific literature has independently explored the relationships between job satisfaction, burnout, and health effects, limitations persist in integrating critical dimensions such as guilt into structural models applied to cabin crew. Under this scenario, it is pertinent to adopt the theoretical framework proposed by Gil-Monte [30], who conceptualizes Burnout Syndrome as a multidimensional process emerging in contexts of high demands and complex interpersonal relationships. Unlike other approaches, this model incorporates the dimension of guilt, which is essential for visualizing how burnout interacts with other variables. This conceptualization is appropriate for job roles characterized by constant high emotional and labor demands with people, as is the case with cabin crew, and represents a relevant contribution to occupational health sciences.

Considering the presented context, this research is developed with the central objective of testing a structural model where Burnout, with its four dimensions (Enthusiasm toward the job, Psychological strain, Indolence, and Guilt), acts as a mediating factor in the relationship between Job Satisfaction and Health Problems among cabin crew in the aviation sector. Integrating this explanatory model allows for a joint understanding of the relationships between these constructs. To understand Gil-Monte’s model, it is relevant to conceptually distinguish between Psychological strain, i.e., the tendency of professionals to evaluate themselves and their work with clients negatively [31], and Indolence, which is the appearance of negative attitudes of indifference and cynicism toward the organization’s clients [32]. Both dimensions fulfill different functions within the erosion process. Based on this, an explanatory model is postulated for a sequence among the studied variables, suggesting that job satisfaction predicts a positive effect on enthusiasm toward the job and an inverse effect on psychological strain. These are detailed through the following hypotheses: H1. Job satisfaction is positively related to enthusiasm toward the job; H2. Job satisfaction is negatively related to psychological strain; H3. There is a negative relationship between enthusiasm toward the job and indolence; H4. Job satisfaction is negatively associated with guilt; H5. Psychological strain is positively related to indolence; H6. Psychological strain is positively related to guilt; H7. Indolence is positively associated with guilt; H8. Guilt is positively related to health problems.

## 2. Materials and Methods

### 2.1. Design

This study is based on a cross-sectional design with a quantitative, descriptive approach within a company in the aviation sector. Participants were cabin crew members selected through non-probability convenience sampling. Data was collected via an electronic questionnaire, ensuring voluntary participation and strict confidentiality. While this sampling method is common in organizational contexts with restricted access, its limitations regarding statistical representativeness and the generalizability of results are acknowledged. Nevertheless, it is appropriate for analyzing relationships between variables and testing the theoretical models that constitute the primary objective of this study.

### 2.2. Characterization of the Sample

A total of *n* = 732 individuals participated in the study, consisting of 204 men (28%) and 528 women (72%). Regarding contract types, 707 (97%) held permanent contracts, while 25 (3%) had fixed-term contracts. Participants had an average professional tenure of 7.27 years. In terms of educational attainment, 4.6% reported postgraduate studies, 46.4% had completed higher education (Bachelor’s level), 22.7% university-level studies, 20.1% technical education, and 6.1% secondary education. These variables were not included as covariates in the structural model to maintain a parsimonious and theoretically focused analysis of the psychosocial constructs.

### 2.3. Instruments

#### 2.3.1. The Spanish Burnout Inventory (SBI)

The CESQT is an instrument validated in organizational settings to measure SQT among workers. This instrument consists of 20 items, with four dimensions: (1) Enthusiasm for Work (5 items): Job satisfaction of workers in achieving their goals. (2) Mental Exhaustion (4 items): The emotional and physical exhaustion of the worker, considering daily contact with people who present causes or problems. (3) Indolence (6 items): Attitude of the worker characterized by cynicism and indifference towards users and their own work environment. Guilt (5 items): Feelings of guilt in the worker, considering their negative attitudes and the work environment.

#### 2.3.2. Job Satisfaction

The UNIPSICO subscale, consisting of six items (α study = 0.77) [33], was used to measure job satisfaction. The job satisfaction subscale determines how workers view their work environment, considering the influence of job satisfaction, decision-making support, and opportunities of interest in their professional development. The Likert scale was used with participants, with five ranks ranging from “Very dissatisfied” to “Very satisfied.”

#### 2.3.3. Psychosomatic Problems

The UNIPSICO subscale of six items (α study = 0.87) [33]. This subscale focuses on assessing the relationship between stress symptoms and psychosocial risks in the work environment.

This subscale measures physical symptoms and psychosomatic disorders with six items, considering physical fatigue, headaches, insomnia, and anxiety that affect work environments. Participants respond using a 5-point Likert scale ranging from “Never” (0) to “Frequently” (4).

### 2.4. Procedure

The procedure was conducted within an international aviation company. The principal investigators contacted the company’s management to obtain authorization for the study. Following approval, the research team reached out to workers to encourage the highest possible participation. Cabin crew members were informed about the study’s purpose, the confidentiality of their responses, and the voluntary nature of the electronic application, which was conducted during their time off to ensure no individual or organizational evaluation consequences. The questionnaire was completed electronically, taking an average of 45 min. Participants were required to read and sign an informed consent protocol before participating.

### 2.5. Data Analysis

Data processing and statistical analysis were performed using the R statistical environment (version 3.6.4) [34]. To evaluate the proposed model, Structural Equation Modeling (SEM) [35] was implemented. This technique was selected for its ability to simultaneously evaluate the interdependent relationships between latent variables. Unlike conventional regression analysis, SEM allows for the integration of measurement error inherent in psychometric instruments, providing more precise trajectory coefficient estimates and enabling the assessment of the global fit of the theoretical model against the empirical data.

The model specification followed a confirmatory approach, strictly based on the hypotheses derived from Gil-Monte’s model. Each latent variable was modeled from its respective observed indicators, assuming uncorrelated measurement errors to preserve parsimony. To ensure the integrity of the deductive process, no post hoc modifications were made based on fit indices, nor were covariances included between errors without theoretical justification; paths not included in the original hypotheses were fixed at zero. Regarding missing data, a mean imputation procedure was used due to the low attrition rate observed. While the limitations of this approach, such as potential variance underestimation, are recognized, the results were interpreted with methodological prudence to ensure the sample’s representativeness was not compromised.

## 3. Results

First, the measurement model was evaluated to examine the psychometric properties of the instruments and the adequacy of the indicators in representing the study’s latent constructs.

Prior to model estimation, basic descriptive statistics were assessed, including standard deviations, skewness, and kurtosis, along with internal consistency indicators for the scales used. These analyses verified the distribution of variables and the internal coherence of the instruments. Given the sample size and the estimation method, the data were found to meet the requirements for the application of Structural Equation Modeling (SEM).

Furthermore, all Cronbach’s Alpha values for the evaluated scales were within acceptable ranges [35] with the lowest value being 0.63 (Indolence/Burnout dimensions) and the highest reaching 0.88 (Enthusiasm toward the job and Psychological strain). Similarly, corrected item–total correlations reached values above 0.40 for most items, contributing to the internal consistency of their respective scales. Specific analyses for convergent or discriminant validity are not reported for this sample as the study focused on evaluating structural relationships between constructs rather than psychometric validation. An initial exploratory analysis was also performed to evaluate missing data patterns and verify normality assumptions.

As predicted, the SEM results showed statistically significant partial associations between the included variables. Specifically, job satisfaction was positively associated with enthusiasm toward the job (H1) and negatively with psychological strain (H2) and guilt (H4). A negative association was also observed between enthusiasm toward the job and indolence (H3). In turn, psychological strain was positively associated with indolence (H5) and guilt (H6), while indolence showed a positive association with guilt (H7). Finally, guilt demonstrated a significant positive association with psychosomatic problems (H8).

### Structural Model

Once the adequacy of the measurement model was established, the structural model was estimated to analyze the hypothesized relationships between latent constructs.

**Figure 1:**
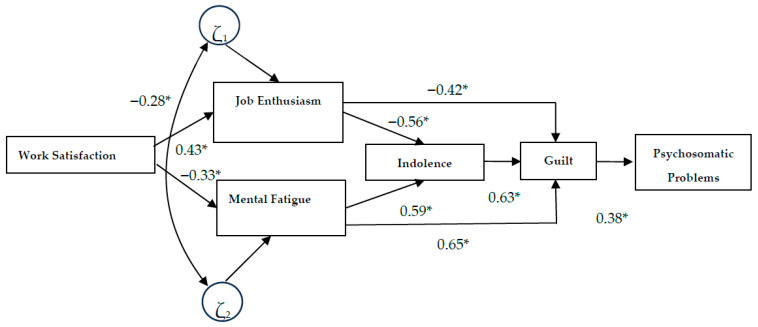
Results of the Structural Equation Model. Note: * = *p* < 0.05.

The model, as described in Figure 1, was evaluated for fit using the Absolute Fit Index (χ^2^), the Tucker–Lewis Index (TLI), the Comparative Fit Index (CFI), and the Root Mean Square Error of Approximation (RMSEA). These indices indicate an acceptable, though not optimal, level of fit for Model 1 (CFI = 0.890; TLI = 0.877; RMSEA = 0.056). Although some indicators fall slightly below the most restrictive cut-off points, these values are considered consistent with a reasonable model fit, particularly given the complexity of the proposed structure and the large sample size. In this regard, several authors have warned that the rigid application of universal cut-off points can be problematic in complex models with large samples, recommending a contextual and substantive interpretation of fit indices rather than a purely normative one [36].

In this model, all relationships between the SBI dimensions and the other variables were significant [37]. From a comparative perspective, the standardized path coefficients suggest that the relationships between psychological strain, indolence, and guilt present higher relative magnitudes within the model. This indicates that these burnout dimensions play a particularly relevant role in the structural dynamics analyzed, while other relationships, though statistically significant, show effects of smaller magnitude.

Consequently, the initial model was consolidated as the final model, considering its standardized paths and error coefficients, as shown in Figure 1. This model highlights the negative relationship with health problems and the positive relationship with enthusiasm toward the job and job satisfaction, presenting an acceptable fit level, χ^2^(1021) = 3856.647, CFI = 0.890, TLI = 0.877, RMSEA = 0.056 (0.051–0.053). These values meet the criteria for evaluating structural models in large samples. The interpretation of these indices took into account the known limitations in large samples, particularly the sensitivity of the χ^2^ statistic to sample size. Therefore, a joint evaluation of multiple indicators was prioritized. In addition to the primary model (Model 1), two additional models (Models 2 and 3) were estimated, and their fit indices are presented in Table 1 for comparative purposes as exploratory alternatives.

## 4. Discussion

The results of this study provide empirical evidence regarding the relationship between job satisfaction, burnout, and health problems in cabin crew, consistent with previous research highlighting the central role of psychosocial processes in high-demand work environments.

First, the findings confirm that job satisfaction acts as a critical antecedent, showing a positive relationship with enthusiasm toward the job (H1) and an inverse relationship with psychological strain (H2). This structure corroborates Gil-Monte’s theoretical model [38], demonstrating that the core dimensions of burnout are articulated in a hierarchical and differentiated manner regarding their impact on health. In this sense, the results not only confirm the direction of the hypothesized associations but also provide relevant information about their relative weight within the model. This aligns with reports by Figueiredo et al. [39] and Ozel et al. [39], who identified similar attitudinal patterns in both technical and cabin crews. The consistency of these data with previous meta-analyses [40,41] reinforces the idea that a positive perception of the work environment not only mitigates exhaustion but also nurtures the worker’s motivational resources.

A fundamental contribution of this research is the identification of specific stressors that erode satisfaction in the aviation sector. Shift irregularity, chronodisruption due to layovers away from home, and the management of high-density flights act as precursors to cognitive and emotional impairment. If these demands become chronic, they trigger the symptoms of physical and mental fatigue that form the basis of burnout [42]. Our results validate the four-factor structure of the Spanish Burnout Inventory (Enthusiasm, Strain, Indolence, and Guilt) in this population, demonstrating the replicability of this psychosocial deterioration process in highly complex operational environments [32,43,44,45].

Particularly revealing is the mediating role of guilt in the relationship between burnout and health problems (H8). The data suggest that among cabin crew, the development of indolence—as a distancing mechanism against passenger demands—often leads to feelings of remorse or guilt. This transition defines Profile 2 of Gil-Monte’s model, where guilt acts as a bridge that exacerbates the worker’s clinical deterioration, linking it to anxiety symptoms and other somatic alterations [46,47]. Consequently, guilt is not a mere adjacent symptom but a determining factor in the syndrome’s severity. These findings underscore the need to design preventive interventions that address not only fatigue but also provide tools for managing emotional labor, preventing the necessary distancing for self-protection from becoming a source of psychological distress and physical pathology.

## 5. Conclusions and Limitations

This study delves into the psychosocial processes associated with burnout in cabin crew, contributing significantly to the literature on this sector. The results highlight the relevance of variables such as indolence, psychological strain, and especially guilt in the manifestation of psychosomatic symptoms and negative work attitudes.

In the analyzed samples, it was found that guilt partially mediates the relationship between indolence and health problems. These effects, both direct and indirect, were statistically significant, reinforcing the hypothesis that emotions like guilt play a key role in work-related psychosocial deterioration. In practical terms, it is suggested that guilt assessment be included as part of the burnout diagnosis in the aviation sector, allowing for a distinction between different burnout profiles. Specifically, the utility of Gil-Monte’s model [38] is highlighted for identifying two profiles: one relatively independent of critical clinical damage (Profile 1) and another with disabling symptomatology (Profile 2). Finally, the need for early interventions, stress management training programs, and specific preventive strategies for cabin crew is emphasized. These actions could improve both individual well-being and organizational functioning, particularly in aviation contexts where occupational stress is high.

Despite these contributions, this study acknowledges several methodological limitations. First, the cross-sectional design precludes establishing clear causal relationships between guilt and psychosomatic disorders; therefore, future longitudinal research is recommended. Practical implications should be interpreted with caution and understood as guidelines rather than prescriptions. Second, the use of self-report questionnaires may have introduced common method variance (CMV) bias. Third, this study focuses exclusively on cabin crew, which restricts the generalizability of the results to other aviation roles. Fourth, the sample consisted solely of Chilean workers. However, the implementation of strategies to mitigate potential biases, such as ensuring response confidentiality, participant unfamiliarity with the evaluated scales, the reverse-coding of certain items, and Harman’s single-factor test [48] (which showed explained variance below the critical threshold), helped control for significant biases. Finally, as the sample comes from a single airline, the findings may not fully represent the entire aviation sector. Consequently, it is necessary to replicate the proposed model in other organizations and operational contexts to evaluate the consistency and transferability of these results.

## Figures and Tables

**Table 1:** Fit Indices for the Resulting Models.

	χ^2^	Df	CFI	TLI	RMSEA (90% IC)	χ^2^/df
Model 1 *	3856.647	1234	0.890	0.877	0.056 (0.051–0.053)	2.98
Model 2	2467.412	762	0.860	0.823	0.052 (0.051–0.050)	3.11
Model 3	3055.562	1098	0.834	0.837	0.050 (0.050–0.051)	3.21

Note: * Although none of the evaluated models met the most stringent global fit thresholds, Model 1 demonstrated a comparatively superior fit relative to the alternative models.

## Data Availability

The data presented in this study are available on request from the second corresponding author, Víctor Olivares-Faúndez, considering the professional ethics and confidentiality indicated to the participants.
